# Influence of dual‐specificity protein phosphatase 5 on mechanical properties of rat cerebral and renal arterioles

**DOI:** 10.14814/phy2.14345

**Published:** 2020-01-20

**Authors:** Huawei Zhang, Chao Zhang, Yedan Liu, Wenjun Gao, Shaoxun Wang, Xing Fang, Ya Guo, Man Li, Ruen Liu, Richard J. Roman, Peng Sun, Fan Fan

**Affiliations:** ^1^ Department of Pharmacology and Toxicology University of Mississippi Medical Center Jackson MS USA; ^2^ Department of Neurosurgery Affiliated Hospital of Qingdao University Qingdao China; ^3^ Department of Neurosurgery Peking University People's Hospital Beijing China

**Keywords:** distensibility, *Dusp5*, elastic modulus, interlobular arterioles, parenchymal arterioles, vascular stiffness

## Abstract

We recently reported that KO of Dual‐specificity protein phosphatase 5 (*Dusp5*) enhances myogenic reactivity and blood flow autoregulation in the cerebral and renal circulations in association with increased levels of pPKC and pERK1/2 in the cerebral and renal arteries and arterioles. In the kidney, hypertension‐related renal damage was significantly attenuated in *Dusp5* KO rats. Elevations in pPKC and pERK1/2 promote calcium influx in VSMC and facilitate vasoconstriction. However, whether DUSP5 plays a role in altering the passive mechanical properties of cerebral and renal arterioles has never been investigated. In this study, we found that KO of *Dusp5* did not alter body weights, kidney and brain weights, plasma glucose, and Hb_A1C_ levels. The expression of pERK is higher in the nucleus of primary VSMC isolated from *Dusp5* KO rats. *Dusp5* KO rats exhibited eutrophic vascular hypotrophy with smaller intracerebral parenchymal arterioles and renal interlobular arterioles without changing the wall‐to‐lumen ratios. These arterioles from *Dusp5* KO rats displayed higher myogenic tones, better distensibility, greater compliance, and less stiffness compared with arterioles from WT control rats. VSMC of *Dusp5* KO rats exhibited a stronger contractile capability. These results demonstrate, for the first time, that DUSP5 contributes to the regulation of the passive mechanical properties of cerebral and renal arterioles and provide new insights into the role of DUSP5 in vascular function, cancer, stroke, and other cardiovascular diseases.

## Graphical abstract

The results from this manuscript demonstrate, for the first time, that DUSP5 contributes to the regulation of the passive mechanical properties of cerebral and renal arterioles and provide new insights into role of DUSP5 in vascular function, cancer, stroke, and other cardiovascular diseases.

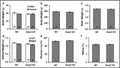

## 1 INTRODUCTION

Dual‐specificity protein phosphatase 5 (DUSP5) inactivates the extracellular signal‐related kinase (ERK1/2) by dephosphorylating threonine/tyrosine residues (Alonso et al., 2004; Kidger & Keyse, 2016; Tonks, 2013). We previously reported that knockout (KO) of *Dusp5* enhances myogenic reactivity and blood flow autoregulation in the cerebral and renal circulations, which is associated with increased levels of phosphorylated protein kinase C (pPKC) and ERK1/2 (pERK1/2) in the cerebral and renal arteries and arterioles (Fan et al., 2014; Zhang et al., 2019). In the kidney, improved hemodynamics in *Dusp5 KO* rats may contribute, at least in part, to the protection from hypertension‐related renal damage (Zhang et al., 2019). The mechanisms by which activation of the PKC and mitogen‐activated protein (MAP)/ERK (MEK) pathways in vascular smooth muscle cells (VSMCs) promotes vasoconstriction involve facilitating calcium influx—by alteration of the activities of multiple ion channels, and enhancing actin–myosin interactions—by modulation of the expression and activities of their associated enzymes and proteins (Zhang et al., 2019).

Activation of PKC and MAP/ERK pathways has been reported to enhance cell proliferation (Chambard, Lefloch, Pouyssegur, & Lenormand, 2007; Gao et al., 2009). Inhibition of DUSP5 expression in human corneal epithelial cells increased ERK1/2 phosphorylation and cell proliferation by 50%–60% (Wang et al., 2010). In *Dusp5* KO rats, we expected that the media of the vascular wall containing VSMCs would be hypertrophied, which would enhance the myogenic response. Surprisingly, although the afferent arterioles (Af‐arts), middle cerebral arteries (MCAs), and renal interlobular arterioles (IAs) of *Dusp5* KO rats exhibited enhanced constrictions in response to elevated transmural pressure, we found these vessels are not larger in calcium‐free media compared with those isolated from wild‐type (WT) control rats (Fan et al., 2014; Zhang et al., 2019).

Changes in the passive mechanical properties of the vascular wall also have a significant influence on myogenic reactivity and blood flow autoregulation. This study investigated the possible role of DUSP5 on vascular mechanical properties by comparing the sizes, incremental distensibility, circumferential wall strain, stress, and the elastic modulus of the intracerebral parenchymal arterioles (PAs) and renal IAs isolated from *Dusp5* KO and WT rats.

## 2 MATERIALS AND METHODS

### 2.1 Animals

Experiments were carried out on 9‐ to 12‐week‐old male *Dusp5* KO and WT rats that we previously generated (Fan et al., 2014; Zhang et al., 2019). All rats were bred and housed at the University of Mississippi Medical Center (UMMC) and were fed a standard diet (Harland) and water ad libitum throughout the studies. All procedures were approved by the Institutional Animal Care and Use Committee of UMMC. All rats related in this project (study rats, breeders, and extra pups that were euthanized) were weighed upon weaning at 3‐week of age, including 38 male and 55 female *Dusp5* KO rats, as well as 60 male and 64 female WT control rats.

### 2.2 Drugs and reagents

All chemicals were purchased from Sigma‐Aldrich. Physiological salt solution (PSS) contained 119 NaCl, 4.7 KCl, 1.17 MgSO_4_, 1.6 CaCl_2_, 18 NaHCO_3_, 5 HEPES, 1.18 NaH_2_PO_4_, and 10 glucose (in mM, pH7.4). Calcium‐free physiological salt solution (PSS_0Ca_) was identical to PSS except for the exclusion of CaCl_2_ and the addition of EDTA (0.03 mM), as we previously described (Fan et al., 2015, 2014, 2017, 2013).

### 2.3 Preparation of arterioles

In the morning on the day of the experiments, plasma glucose and Hb_A1C_ were measured using a Contour Next Meter System (Fisher Scientific, Waltham, MA) and Polymer Technology Systems A1CNow+™ Systems (Fisher Scientific) according to the manufacturer's instructions. The rats were then euthanized with 4% isoflurane and weighed. The brains and kidneys were collected, weighed, and placed in a dish filled with ice‐cold PSS_0Ca_. A piece of the brain surrounding the MCA was removed and transferred to another dish filled with ice‐cold PSS_0Ca_ supplemented with 1% bovine serum albumin (BSA) for vascular dissection. PAs branching directly from the M1 segment of MCA were carefully dissected (Cipolla, Chan, et al., 2014a; Cipolla, Sweet, et al., 2014b; Pires, Dabertrand, & Earley, 2016) under a microscope and mounted on glass micropipettes in a chamber filled with warmed (37°C), oxygenated (21% O_2_, 5% CO_2_, and 74% N_2_) PSS.

The kidneys were cut into 1‐mm‐thick slices, and a piece of the cortex was transferred to a dish filled with ice‐cold PSS_0Ca_ containing 1% BSA. Renal IAs upstream of Af‐arts were dissected under a stereomicroscope and mounted on glass micropipettes in a chamber filled with warmed and oxygenated PSS.

### 2.4 Pressure myography

Intact cerebral PAs and renal IAs isolated from *Dusp5* KO and WT rats were mounted on glass cannulas in a pressure myography chamber (Living System Instrumentation) mounted on an IMT‐2 inverted microscope (Olympus). The pressure was initially set to 10 mmHg for PAs and 60 mmHg for IAs and vessels were equilibrated for 30 min to generate a spontaneous tone. Inner and outer diameters (ID and OD) in response to increases in intraluminal pressures (from 10 to 60 mmHg for PAs and 60 to 180 mmHg for IAs) were measured using a digital camera attached to the microscope. Perfusion pressure was slowly increased from 10 to 60 mmHg for PAs in 10 mmHg increments. For IAs, intraluminal pressure was slowly raised from 60 to 180 mmHg in a stepwise fashion.

At the end of the experiment, intraluminal pressure was reset to 5 mmHg, and the vessels were washed with PSS_0Ca_ for 6–8 times. Inner and outer diameters under calcium‐free conditions (ID_0Ca_ and OD_0Ca_) of these arterioles were determined at 5 mmHg, 10–60 mmHg for PAs, and 60–180 mmHg for IAs as described above.

### 2.5 Calculation of structure parameters

The following vascular mechanical properties were calculated using equations described previously (Baumbach, Heistad, & Siems, 1989; Briones et al., 2003; Cheng et al., 2014; Dobrin, 1978; Gonzalez et al., 2005; Hudetz, 1979; Izzard et al., 2003):

$$\text{Wall thickness}\left(\mu m\right)=\left({\text{OD}}_{0\text{Ca}}-{\text{ID}}_{0\text{Ca}}\right)/2$$

$$\text{Cross}-\text{sectional area}\left(\text{CSA},\mu m^{2}\right)=\left(\pi /4\right)\times \left({\text{OD}}_{0\text{Ca}}^{2}-{\text{ID}}_{0\text{Ca}}^{2}\right).$$

$$\text{Wall to lumen ratio}=\text{Wall thickness}/{\text{ID}}_{0\text{Ca}}$$

$$\text{Myogenic tone}\left(\%\right)=\left[\left({\text{ID}}_{0\text{Ca}}-\text{ID}\right)/{\text{ID}}_{0\text{Ca}}\right]\times 100.$$

$$\text{Distensibility}\left(\%\right)=\left({\text{ID}}_{0\text{Ca}}-{\text{ID}}_{0\text{Ca}\;5\text{mmHg}}\right)/{\text{ID}}_{0\text{Ca}\;5\text{mmHg}}\times 100.$$

where ID_0Ca 5mmHg_ is inner diameters obtained at the perfusion pressure of 5 mmHg in PSS_0Ca_.

$$\begin{array}{c} \text{Incremental distensibility}\left(\%/\text{mmHg}\right)=\Delta {\text{ID}}_{0\text{Ca}}/\left({\text{ID}}_{0\text{Ca}}\times \Delta P\right)\\ \;\;\;\;\;\;\;\;\;\times 100. \end{array}$$

Incremental distensibility defines as the percentage change in the vascular ID_0Ca_ for every 1 mmHg changes in P. Where P is the intraluminal pressure in PSS_0Ca_.

The circumferential wall strain defined as

$$\text{Circumferential wall strain}\left(\varepsilon \right)=\left({\text{ID}}_{0\text{Ca}}-{\text{ID}}_{0\text{Ca}\;5\text{mmHg}}\right)/{\text{ID}}_{0\text{Ca}\;5\text{mmHg}}$$

$$\text{Circumferential wall stress}\left(\sigma \right)=\left(P\times {\text{ID}}_{0\text{Ca}}\right)/2\times \text{wall thickness}$$

where P indicates intraluminal pressure (1 mmHg = 133.4 Nm^‐2^) under calcium‐free conditions.

Arterial stiffness refers to the ability of arteries to resist elastic deformation when subjected to pressure. It is determined by elastic modulus (*E* = *σ/ε*). This relationship is non‐linear and appropriate to the exponential curve. Thus, an exponential model with least‐squares analysis was used:

$$\sigma =\sigma_{\text{orig}}e^{\beta \varepsilon }$$

where *σ*_orig_ is *σ* at the original diameter at 5 mmHg. The slope of the curve (*β* value) was used to determine the tangential or incremental elastic modulus (*E*_inc_), which is directly proportional to *E*_inc_. An increased *β* value indicated increases in stiffness.

### 2.6 Isolation of vascular smooth muscle cells

Primary VSMCs were isolated from WT (*n* = 3) and *Dusp5* KO (*n* = 3) rats, as described previously (Fan et al., 2017). Briefly, the cerebral and renal vessels were isolated using the Evans blue sieving procedure (Fan et al., 2015, 2013), and digested with dithiothreitol (2 mg/ml, Sigma‐Aldrich), papain (22.5 U/mL, Sigma‐Aldrich), trypsin inhibitor (10,000 U/mL, Sigma‐Aldrich), collagenase (250 U/mL, Sigma‐Aldrich), and elastase (2.4 U/mL, Sigma‐Aldrich) at 37°C. After centrifugation, the VSMCs were resuspended and seeded on autoclaved glass coverslips precoated with CellTak (Thermo Scientific) in a six‐well plate. Early passages (P2‐P3) of the primary VSMCs were used for the following experiments.

### 2.7 Immunocytochemistry

Primary VSMCs were fixed with 3.7% paraformaldehyde (Thermo Scientific), and permeabilized with 0.1% Triton‐100 (Sigma‐Aldrich), and blocked with 1% BSA. The cells were then incubated with the primary antibody p44/42 MAPK (ERK1/2; 1:50, #4696, Cell Signaling) or phosphor‐p44/42 MAPK (pERK1/2; 1:100, #4377, Cell Signaling), following by Alexa Fluor 555 or Alexa Fluor 488–labeled secondary antibodies (Thermo Scientific). The slides were coverslipped after dropping an anti‐fade mounting medium with DAPI (Vector Laboratories). Images were captured using a Nikon C2 confocal microscope (Nikon), and the mean fluorescence intensities per cell were compared using NIS‐Elements Imaging Software 4.6 (Nikon). Experiments were repeated three times, and triplicate wells were used at each experiment.

### 2.8 Cell constriction assay

The contractile capability of primary VSMCs isolated from WT versus *Dusp5* KO was compared using a collagen gel‐based assay kit (Cell Biolabs) following manufactory instruction. Briefly, the VSMCs (2 × 10^6^ cells/mL) were suspended in the culture medium, mixed with collagen gel working solution, added in a 24‐well plate, and incubated at 37°C for 2 days to develop contractile stress. The cell contraction was initiated by detaching the stressed matrix from the wall of the culture plate using a sterile needle. Percentage changes in the size of the collagen gel were imaged at 30 min interval and analyzed using the NIS‐Elements Imaging Software 4.6 (Nikon).

### 2.9 Statistical analysis

Data are presented as mean values ± standard error (*SEM*). The differences in the means between groups and the slopes of stress–strain curves were compared using Student's *t* test. The significance of differences in pressures–diameter relationships was analyzed using an analysis of variance (ANOVA) for repeated measures with multiple groups followed by a Holm‐Sidak post hoc test using GraphPad Prism 6 (GraphPad Software, Inc.). A value of *p* < .05 was considered to be significant.

## 3 RESULTS

### 3.1 Effects of knockout of Dusp5 on body weights, brain and kidney weights, plasma glucose, and Hb_A1C_ levels

**Figure 1:**
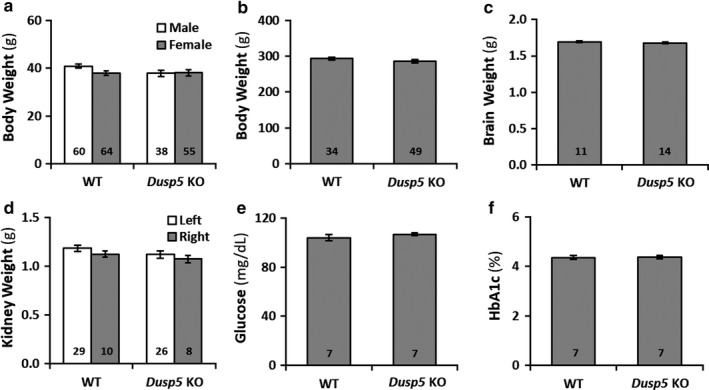
Effects of Knockout of Dual‐specificity protein phosphatase 5 (*Dusp5*) on body weights, brain and kidney weights, plasma glucose and Hb_A1C_ levels. (a) Comparison of body weights in 3‐week‐old male and female *Dusp5* KO versus WT rats. (b) Comparison of body weights in 9‐ to 12‐week‐old male *Dusp5* KO versus WT rats. (c) Comparison of brain weights in 9‐ to 12‐week‐old male *Dusp5* KO versus WT rats. (d) Comparison of kidney weights in 9‐ to 12‐week‐old male *Dusp5* KO versus WT rats. (e) Comparison of plasma glucose levels in 9‐ to 12‐week‐old male *Dusp5* KO versus WT rats. (f) Comparison of Hb_A1C_ levels in 9‐ to 12‐week‐old male *Dusp5* KO versus WT rats. Mean values ± *SEM* are presented. Numbers indicate the number of animals studied per group

As presented in Figure 1a, there were no differences in body weight in both sexes of *Dusp5* KO (males: 37.82 ± 1.39 g, *n* = 38; females: 38.05 ± 1.30 g, *n* = 55) versus WT (males: 40.82 ± 0.80 g, *n* = 60; females: 37.86 ± 0.99 g, *n* = 64) rats when they were 3‐week of age. Similarly, body weight was not different in male *Dusp5* KO (285.79 ± 4.52 g, *n* = 34) versus WT (293.16 ± 3.44 g, *n* = 49) rats when they were at 9–12 weeks of age (Figure 1b). Brain weight was not significantly different between *Dusp5* KO (1.68 ± 0.01 g, *n* = 14) and WT (1.70 ± 0.01 g, *n* = 11) rats (Figure 1c). There were no differences in kidney weight between *Dusp5* KO (left kidney: 1.12 ± 0.04 g, *n* = 26; right kidney: 1.07 ± 0.04 g, *n* = 8) and WT (left kidney: 1.19 ± 0.03 g, *n* = 29; right kidney: 1.12 ± 0.03 g, *n* = 10) rats (Figure 1d). Plasma glucose (Figure 1e) and Hb_A1C_ (Figure 1f) levels were similar in 9‐ to 12‐week‐old *Dusp5* KO (106.86 ± 1.10 mg/dl, *n* = 7; and 4.37 ± 0.06%, *n* = 7, respectively) versus WT (104.00 ± 2.51 mg/dl, *n* = 7; and 4.36 ± 0.08%, *n* = 7, respectively) rats.

### 3.2 Effects of knockout of Dusp5 on the expression and localization of ERK and pERK in primary VSMCs

**Figure 2:**
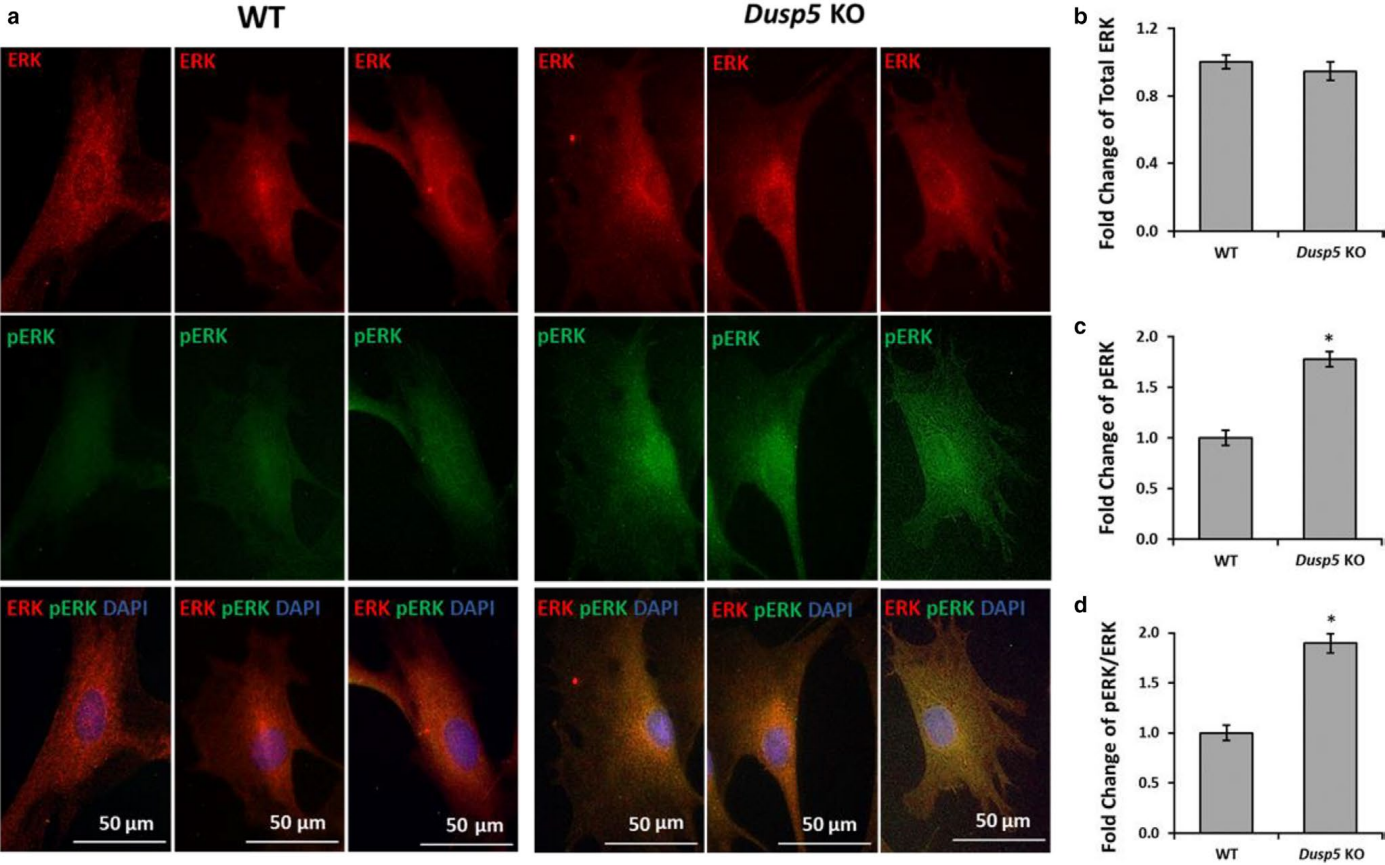
Effects of knockout of Dual‐specificity protein phosphatase 5 (*Dusp5*) on the expression and localization of ERK and pERK in primary VSMCs. (a) Representative images of the expression and localization of total ERK and pERK in primary VSMCs isolated from WT and *Dusp5* KO rats. (b) Quantitative analysis of fold changes in the mean red fluorescence intensity in VSMC of *Dusp5* KO versus WT rats. (c) Quantitative analysis of fold changes in the mean green fluorescence intensity in VSMC of *Dusp5* KO versus WT rats. (d) Quantitative analysis of fold changes in pERK/ERK in VSMC of *Dusp5* KO versus WT rats. Primary VSMCs were isolated from three rats of each strain. Experiments were repeated three times, and triplicate wells were used at each experiment. * indicates *p* < .05 from the corresponding value in *Dusp5* KO versus WT rats

The expression of total ERK was similar in the nucleus and cytoplasm in primary VSMCs isolated from WT and *Dusp5* KO rats (Figure 2a,b). However, the expression of pERK was higher in the nucleus of primary VSMCs isolated from *Dusp5* KO rats (Figure 2a,c). The ratio of pERK/ERK was increased by 1.9 ± 0.1 folds (Figure 2d) in *Dusp5* KO versus WT rats.

### 3.3 Effects of knockout of Dusp5 on vascular characteristics of cerebral PAs

**Figure 3:**
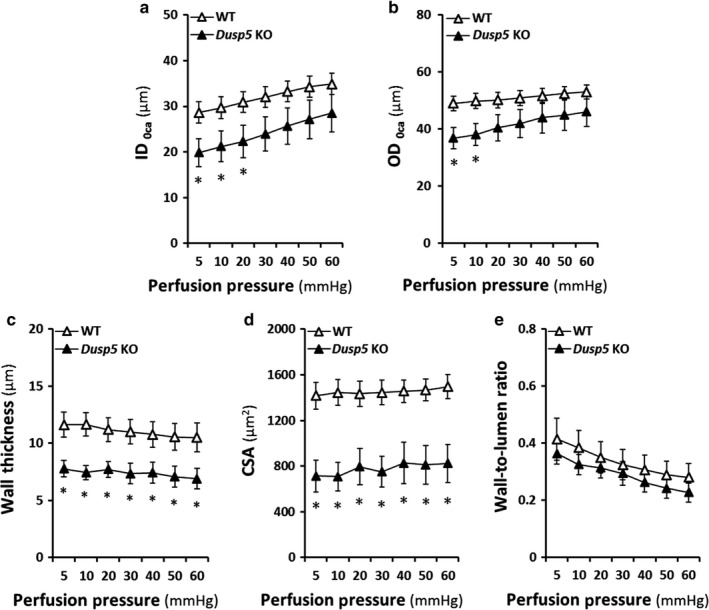
Effects of Knockout of Dual‐specificity protein phosphatase 5 (*Dusp5*) on vascular characteristics of cerebral PAs. (a) Comparison of ID_0Ca_ of PAs of *Dusp5* KO versus WT rats. (b) Comparison of OD_0Ca_ of PAs of *Dusp5* KO versus WT rats. (c) Comparison of wall thicknesses of PAs of *Dusp5* KO versus WT rats. (d) Comparison of cross‐sectional areas (CSA) of PAs of *Dusp5* KO versus WT rats. (e) Comparison of the wall‐to‐lumen ratios of PAs of *Dusp5* KO versus WT rats. All rats studied were 9‐ to 12‐week‐old males. Mean values ± *SEM* are presented. *N* = 4–8 rats per group. * indicates *p* < .05 from the corresponding value in *Dusp5* KO versus WT rats

Figure 3 demonstrates a comparison of vascular characteristics of cerebral PAs between *Dusp5* KO versus WT rats. ID_0Ca_ (19.87 ± 2.2 μm) and OD_0Ca_ (36.84 ± 3.7 μm) of PAs were smaller in *Dusp5* KO rats at 5 mmHg intraluminal pressure than WT (28.63 ± 2.0 μm and 49.98 ± 2.5 μm, respectively). These differences remained at low pressures but were diminished at higher perfusion pressures (Figure 3a,b). Wall thickness and CSA of PAs were smaller in *Dusp5* KO versus WT rats at perfusion pressures from 10 to 60 mmHg (Figure 3c,d), and there were no differences in the wall‐to‐lumen ratios between two strains at perfusion pressures from 5 to 60 mmHg (Figure 3e).

### 3.4 Effects of knockout of Dusp5 on vascular characteristics of renal IAs

**Figure 4:**
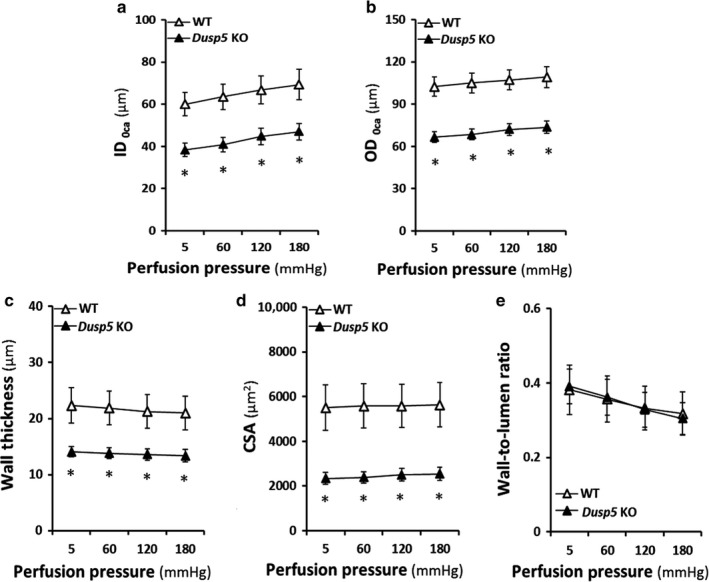
Effects of Knockout of Dual‐specificity protein phosphatase 5 (*Dusp5*) on vascular characteristics of cerebral IAs. (a) Comparison of ID_0Ca_ of IAs of *Dusp5* KO versus WT rats. (b) Comparison of OD_0Ca_ of IAs of *Dusp5* KO versus WT rats. (c) Comparison of wall thicknesses of IAs of *Dusp5* KO versus WT rats. (d) Comparison of cross‐sectional areas (CSA) of IAs of *Dusp5* KO versus WT rats. (e) Comparison of the wall‐to‐lumen ratios of IAs of *Dusp5* KO versus WT rats. All rats studied were 9‐ to 12‐week‐old males. Mean values ± *SEM* are presented. *N* = 8–11 rats per group. * indicates *p* < .05 from the corresponding value in *Dusp5* KO versus WT rats

As presented in Figure 4, vascular characteristics of renal IAs between *Dusp5* KO versus WT rats were compared. ID_0Ca_ (38.29 ± 3.2 μm) and OD_0Ca_ (66.45 ± 3.8 μm) of IAs were smaller in *Dusp5* KO rats at 5 mmHg intraluminal pressure than WT (59.98 ± 5.5 μm and 102.44 ± 6.8 μm, respectively). These differences remained at perfusion pressure from 60 to 180 mmHg. The wall thickness and CSA of IAs were smaller in *Dusp5* KO rats at perfusion pressures from 5 to 180 mmHg (Figure 4a,d). However, there were no differences in the wall‐to‐lumen ratios of IAs in both strains (Figure 4e).

### 3.5 Effects of knockout of Dusp5 on the myogenic tone of cerebral PAs and renal IAs

**Figure 5:**
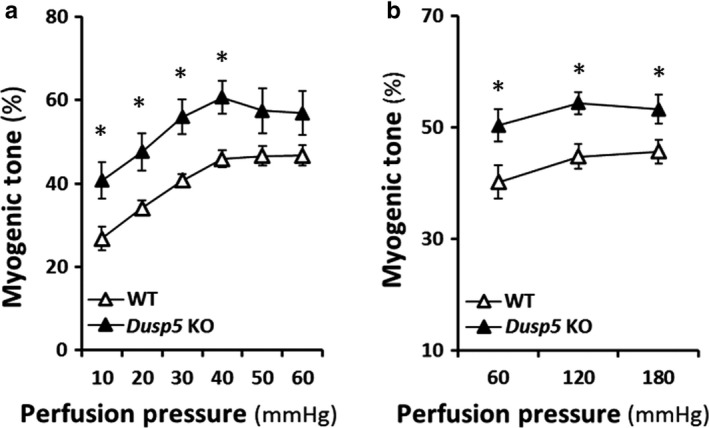
Effects of Knockout of Dual‐specificity protein phosphatase 5 (*Dusp5*) on the myogenic tone of cerebral PAs and renal IAs. (a) Comparison of the myogenic tone of PAs of *Dusp5* KO versus WT rats. (b) Comparison of the myogenic tone of IAs of *Dusp5* KO versus WT rats. All rats studied were 9‐ to 12‐week‐old males. Mean values ± *SEM* are presented. *N* = 4–11 rats per group. * indicates *p* < .05 from the corresponding value in *Dusp5* KO versus WT rats

The myogenic tone of cerebral PAs and renal IAs isolated from *Dusp5* KO versus WT rats is presented in Figure 4. *Dusp5* KO rats exhibited a higher active tone in PAs at perfusion pressures from 10 to 40 mmHg (Figure 5a) and in IAs at intraluminal pressures from 60 to 180 mmHg (Figure 5b).

### 3.6 Effects of knockout of Dusp5 on vascular distensibility and incremental distensibility of cerebral PAs and renal IAs

**Figure 6:**
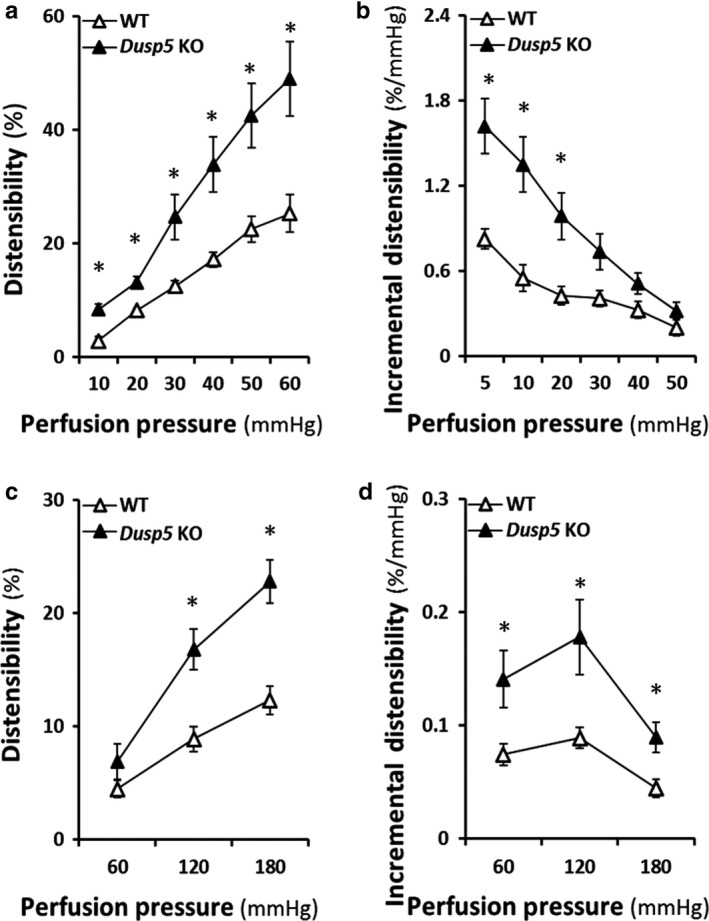
Effects of Knockout of Dual‐specificity protein phosphatase 5 (*Dusp5*) on vascular distensibility and incremental distensibility of cerebral PAs and renal IAs. (a) Comparison of the distensibility of PAs of *Dusp5* KO versus WT rats. (b) Comparison of the incremental distensibility of PAs of *Dusp5* KO versus WT rats. (c) Comparison of the distensibility of IAs of *Dusp5* KO versus WT rats. (d) Comparison of the incremental distensibility of IAs of *Dusp5* KO versus WT rats. All rats studied were 9‐ to 12‐week‐old males. Mean values ± *SEM* are presented. *N* = 4–11 rats per group. * indicates *p* < .05 from the corresponding value in *Dusp5* KO versus WT rats

*Dusp5* KO rats exhibited better distensibility in PAs at perfusion pressures from 10 to 60 mmHg (Figure 6a), and greater incremental distensibility in PAs at perfusion pressures from 10 to 30 mmHg (Figure 6b). Similarly, *Dusp5* KO rats exhibited better distensibility and incremental distensibility in IAs at perfusion pressures from 120 to 180 mmHg (Figure 6c) and from 120 to 180 mmHg (Figure 6d).

### 3.7 Effects of knockout of Dusp5 on elastic modulus and vascular stiffness of cerebral PAs and renal IAs

**Figure 7:**
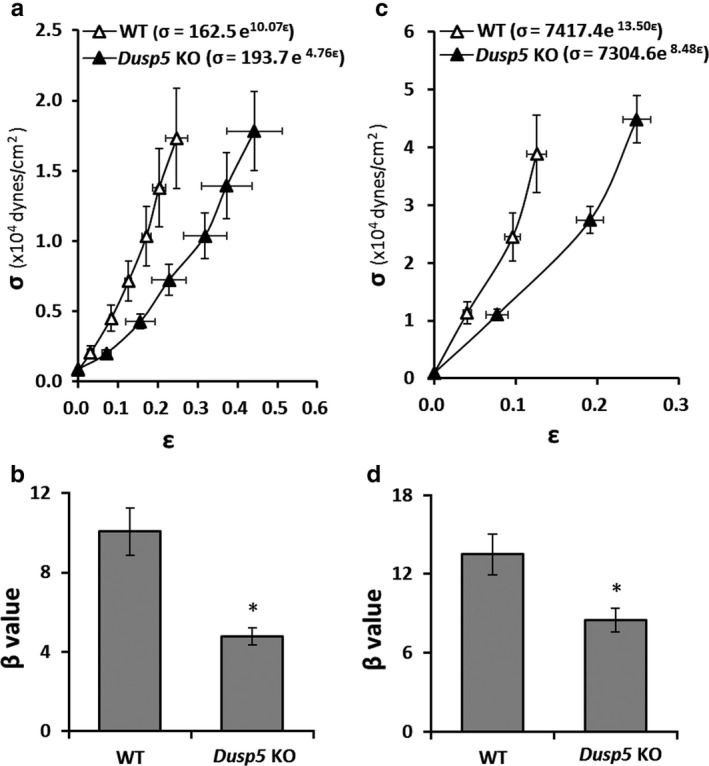
Effects of Knockout of Dual‐specificity protein phosphatase 5 (*Dusp5*) on the elastic modulus and vascular stiffness of cerebral PAs and renal IAs. (a) Comparison of the elastic modulus (stress–strain relationships) of PAs of *Dusp5* KO versus WT rats. (b) Comparison of the slopes of the elastic modulus curves (β value) of PAs of *Dusp5* KO versus WT rats. (c) Comparison of the stress–strain relationships of IAs of *Dusp5* KO versus WT rats. (d) Comparison of the β values of IAs of *Dusp5* KO versus WT rats. All rats studied were 9‐ to 12‐week‐old males. Mean values ± *SEM* are presented. *N* = 4–11 rats per group. * indicates *p* < .05 from the corresponding value in *Dusp5* KO versus WT rats

The stress–strain relationships or the elastic modulus curves of PAs between *Dusp5* KO (*R* ^2^ = 0.95 ± 0.008, *n* = 6) versus WT (*R* ^2^ = 0.98 ± 0.005, *n* = 6) rats were compared (Figure 7a). The β value was significantly smaller in PAs of *Dusp5* KO (4.76 ± 0.43) than in WT (10.07 ± 1.9) rats (Figure 7b). Similarly, by comparison of an elastic modulus curve of IAs (Figure 7c) between *Dusp5* KO (*R* ^2^ = 0.98 ± 0.008, *n* = 8) versus WT (*R* ^2^ = 0.99 ± 0.004, *n* = 8) rats, we found that the β value was significantly smaller in IAs isolated from *Dusp5* KO (8.48 ± 0.088) than WT (13.50 ± 1.54) rats (Figure 7d).

### 3.8 Effects of knockout of Dusp5 on VSMC contractile capability

**Figure 8:**
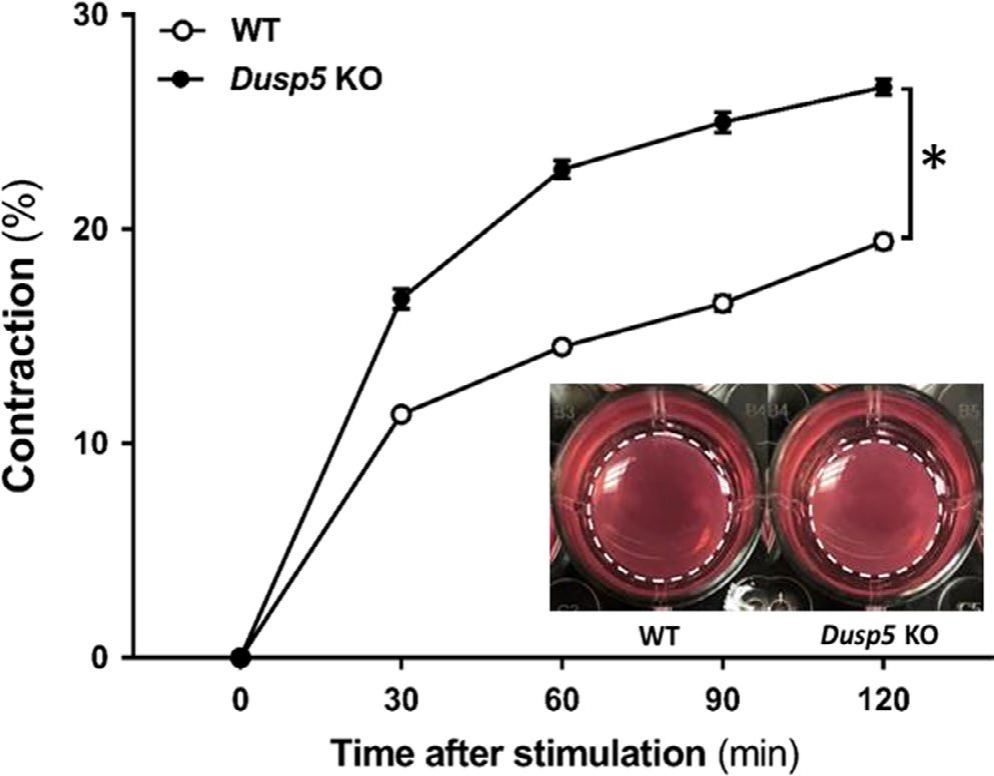
Effects of knockout of Dual‐specificity protein phosphatase 5 (*Dusp5*) on VSMC contractile capability. Comparison of the contractile capability of primary VSMC isolated from the vasculature of *Dusp5* KO versus WT rats. Representative images after 120 min stimulation are presented as the insertion. Experiments were repeated three times, and triplicate wells were used at each experiment. * indicates *p* < .05 from the corresponding value in *Dusp5* KO versus WT rats

The effects of knockout of *Dusp5* on VSMC contractile capability are presented in Figure 8. The VSMCs isolated from the vasculature of *Dusp5* KO rats exhibited a stronger contractile capability, and the gel size after 120 min of stimulation was maximally reduced by 26.6 ± 0.4% versus19.4 ± 0.3% compared with cells isolated from WT rats.

## 4 DISCUSSION

DUSP5 is a nuclear protein, and it dephosphorylates the threonine/tyrosine residues of ERK1/2 to affect numerous cellular functions, which contributes to the pathogenesis in many diseases (Lake, Correa, & Muller, 2016; Mandl, Slack, & Keyse, 2005; Seternes, Kidger, & Keyse, 2019; Zhang et al., 2019). We previously reported that the myogenic response and autoregulation of cerebral and renal blood flow (CBF and RBF) are enhanced in *Dusp5* KO rats (Fan et al., 2014; Zhang et al., 2019). In this study, we compared the passive mechanical properties of intracerebral PAs and renal IAs isolated from *Dusp5* KO and WT rats. The results from this study are as follows: (1) there was no difference in body weights, kidney and brain weights, plasma glucose, and Hb_A1C_ levels between *Dusp5* KO and WT rats. (2) The expression of pERK is higher in the nucleus of primary vascular smooth muscle cells isolated from *Dusp5* KO rats. (3) Inner and outer diameters of PAs and IAs were smaller in *Dusp5* KO versus WT rats. However, the wall‐to‐lumen ratios of these vessels were not significantly different. (4) *Dusp5* KO rats exhibited higher myogenic tones in both PAs and IAs. (5) The incremental distensibility of PAs and IAs were greater in *Dusp5* KO than WT rats. (6) PAs and IAs isolated from *Dusp5* KO rats displayed greater compliance and less stiffness indicating by higher distensibility and incremental distensibility and lower β values. (7) VSMC of *Dusp5* KO rats exhibited a stronger contractile capability.

Arterioles are high resistance vessels in vascular beds. The most significant changes in blood pressure and blood flow occur at the transition of arterioles to capillaries indicating these arterioles play a major role in blood flow autoregulation (Martinez‐Lemus, 2012). The vascular smooth muscle cells (VSMCs) in the tunica media of the arteriolar wall are essential for the myogenic response to regulate blood flow (Fan et al., 2017). In the tunica intima layer, endothelial cells participate in the control of vascular permeability and reactivity by releasing vasoactive factors. Collagen in the intima adventitia and media layers and elastin in the intima and media contribute to the regulation of flexibility and stiffness of the vasculature (Martinez‐Lemus, 2012). In the brain, the large extracranial vessels (internal carotid and vertebral) and intracranial pial vessels provide ~50% of cerebral vascular resistance as determined using a direct measurement of the pressure gradient across different segments of the cerebral circulation (Faraci & Heistad, 1990; Heistad, Marcus, & Abboud, 1978). It has been traditionally thought that small pial, penetrating, and parenchymal arterioles account for the remainder of autoregulation of CBF and the fine regulation of capillary pressure (Federico et al., 2012; Iadecola & Davisson, 2008). In this study, the intracerebral PAs we used are the lenticulostriate branches of the MCAs, which provide blood supply to the frontoparietal white matter tracts and the basal ganglia in rats (Johnson & Cipolla, 2018; Pires et al., 2016). Embolism of these vessels is the most common cause of ischemic stroke in humans (Navarro‐Orozco & Sanchez‐Manso, 2019). We also studied the IAs, which are the corresponding resistance arterioles in the kidney. Resistance along the IAs and Af‐arts accounts for the majority of the preglomerular pressure drop in the renal circulation and plays a major role in RBF autoregulation that protects fragile glomerular capillaries from elevations in systemic pressure (Imig, Zou, Ortiz de Montellano, Sui, & Roman, 1994).

We found there were no differences in body weights, brain, and kidney weights between 12‐week‐old *Dusp5* KO and WT control rats. The inner and out diameters, wall thickness, and cross‐sectional areas of PAs and IAs were smaller in *Dusp5* KO compared with WT rats. This finding is unexpected as the activation of the PKC and ERK pathways is thought to enhance cell proliferation (Chambard et al., 2007; Gao et al., 2009). Inhibition of DUSP5 expression in human corneal epithelial cells increased ERK1/2 phosphorylation, and cell proliferation by 50%–60% (Wang et al., 2010). *Dusp5* KO mice enhanced ERK activity in eosinophils by upregulation of antiapoptotic BCL‐X_L_ and prolonged eosinophil lifespan (Holmes, Yeh, Yan, Xu, & Chan, 2015). However, there is certainly no evidence that loss of DUSP5 alone causes an increase in cell proliferation in either skin cancer or in mouse embryo fibroblasts in *Dusp5* KO mice (Rushworth et al., 2014). Our previous results also demonstrated that 10‐week‐old *Dusp5* KO rats had similar inner diameters of Af‐art, and the sizes of MCAs were not different in 9–12 weeks KO compared with WT controls, interestingly, IAs were smaller in the KO rats when they were 24 weeks old (Fan et al., 2014; Zhang et al., 2019).

DUSP5 has a half‐life of 45 min and can be rapidly degraded by the proteasome (Kucharska, Rushworth, Staples, Morrice, & Keyse, 2009). In this study, we found that the expression of pERK is higher in the nucleus of primary VSMC isolated from *Dusp5* KO rats. In the nucleus, DUSP5 not only inactivates ERK1/2 but also anchors ERK1/2, which was reported to paradoxically enhance cytoplasmic ERK activity in cancer via reduced ERK‐mediated RAF inhibition (Bellou et al., 2009; Kidger & Keyse, 2016; Kidger et al., 2017). On the other hand, activation of cytoplasmic ERK also facilitates nuclear translocation (Kidger et al., 2017; Mebratu & Tesfaigzi, 2009). Cytoplasmic ERK is anchored by an associated phosphatase, MEK, and microtubules (Fukuda, Gotoh, & Nishida, 1997; Reszka, Seger, Diltz, Krebs, & Fischer, 1995). Nuclear ERK activation promotes cell proliferation by enhancing cell cycle regulatory protein activities and posttranslational modifications to increase prosurvival gene function and reduce cell death (Mebratu & Tesfaigzi, 2009). ERK activation also has been reported to play a role in cell death. For example, ERK activation induced by DNA damage agents, IFNγ, or Fas causes cell death or apoptosis. However, the underlying mechanisms remain poorly understood, and the evidence to support this view is less well studied (Mebratu & Tesfaigzi, 2009). Nevertheless, ERK1/2 activation can involve both cell proliferation or apoptosis, depending on its localization, cell type, and physiological and pathological conditions that may involve different sets of ERK effectors (Mebratu & Tesfaigzi, 2009). A good example is that the expression of BCL‐X_L_ has no changes in *Dusp5* KO mice, using a microarray (Rushworth et al., 2014), although it was upregulated in eosinophils in another study (Holmes et al., 2015). Other mechanisms involved in the role of DUSP5 on cell proliferation or apoptosis also cannot be excluded, as this protein has tumor activator or suppressor function in different types of cancers (Montero‐Conde et al., 2013; Pratilas et al., 2009; Rushworth et al., 2014; Shin, Park, & Kang, 2013; Ueda, Arakawa, & Nakamura, 2003; Wang et al., 2019; Yun et al., 2009).

An interesting previous study (Fu, McKnight, Yu, Callaway, & Lane, 2006) demonstrated that intrauterine growth retardation reduced hepatic DUSP5 and enhanced phosphorylation of ERK1/2 and the insulin receptor substrate‐1 (IRS‐1), which contributes to insulin resistance (Fu et al., 2006). We found there was no difference in BW between *Dusp5* KO and WT control rats at 3 and 9–12 weeks of age. In addition, plasma glucose and Hb_A1C_ levels were similar and in the normal ranges in 9–12 weeks old *Dusp5* KO in comparison with WT control rats, suggesting KO of *Dusp5* unlikely induces insulin resistance or hyperglycemia in this study.

Alteration of vessel sizes can change the proportion of components of the vascular wall, which determine the mechanical properties that influence the response of the myogenic reactivity and autoregulation. In this study, the size of PAs and IA was smaller in *Dusp5* KO compared with WT rats. This was associated with increased distensibility and incremental distensibility in cerebral PAs and renal IAs of *Dusp5* KO versus WT rats. The elastic modulus curves were shifted to the right, and the slopes or β values were smaller in the vessels isolated from *Dusp5* KO than WT rats. These results suggest that arterioles in *Dusp5* KO are more compliant and distensible with less stiffness than WT control rats.

It has been reported that *Dusp5* is a vascular endothelial‐specific gene in zebrafish (Pramanik et al., 2009; Qian et al., 2005; Sumanas, Jorniak, & Lin, 2005) and humans (Alleboina et al., 2019). DUSP5 is expressed in angioblasts, and it plays an essential role in embryonic vascular development. Downregulation of this protein promotes endothelial apoptosis in zebrafish (Pramanik et al., 2009) but not in humans (Alleboina et al., 2019). In mice, knockdown of DUSP5 impaired postischemic angiogenesis in association with increased limb necrosis (Alleboina et al., 2019). We have reported that DUSP5 is expressed in renal and cerebral vasculatures in rats; however, the vascular cell specificity of expression of DUSP5 has not been well studied (Fan et al., 2014; Zhang et al., 2019). Arteriolar intrinsic passive mechanical properties influence vascular elasticity and stiffness that have major effects on stretch and shear stress‐induced NO production by vascular endothelial cells (Sriram et al., 2012). On the other hand, the myogenic response, as an intrinsic property of VSMCs, is modulated by the endothelia under different genetic, physiological, and pathological conditions by releasing various vasoactive factors (Fan et al., 2016; Fan & Roman, 2017). Although we found that VSMC of Dusp5 KO rats exhibited a stronger contractile capability, which is consistent with our previous findings that the myogenic response is enhanced in *Dusp5* KO rats, the underlying mechanisms are still not elucidative with regards to how DUSP5 enhances myogenic response and blood flow autoregulation demonstrated in our previous studies (Fan et al., 2014; Zhang et al., 2019). The observations in the current studies provide a piece of new information to better understand the potential mechanisms.

In summary, this study first time demonstrates that DUSP5 contributes to the regulation of passive mechanical properties of cerebral and renal arterioles. KO of *Dusp5* did not alter body and organ (brain and kidney) weights and did not induce insulin resistance or hyperglycemia. *Dusp5* KO rats exhibited eutrophic vascular hypotrophy in intracerebral parenchymal arterioles and renal interlobular arterioles. These arterioles of *Dusp5* KO rats displayed higher myogenic tones, greater distensibility and compliance, and less stiffness compared with arterioles of WT control rats. These results provide new insights into role of DUSP5 in vascular development, cancer, stroke, and other cardiovascular diseases.

## CONFLICT OF INTEREST

None.
